# A Pentasymmetric Open Channel Blocker for Cys-Loop Receptor Channels

**DOI:** 10.1371/journal.pone.0106688

**Published:** 2014-09-03

**Authors:** Valentina Carta, Michael Pangerl, Roland Baur, Roshan Puthenkalam, Margot Ernst, Dirk Trauner, Erwin Sigel

**Affiliations:** 1 Institute of Biochemistry and Molecular Medicine, University of Bern, Bern, Switzerland; 2 Department of Chemistry, Ludwig-Maximilians-Universität München and Center of Integrated Protein Science, Munich, Germany; 3 Department of Biochemistry and Molecular Biology, Center for Brain Research, Medical University of Vienna, Vienna, Austria; Weizmann Institute of Science, Israel

## Abstract

γ-Aminobutyric acid type A receptors (GABA_A_ receptors) are chloride ion channels composed of five subunits, mediating fast synaptic and tonic inhibition in the mammalian brain. These receptors show near five-fold symmetry that is most pronounced in the second trans-membrane domain M2 lining the Cl^−^ ion channel. To take advantage of this inherent symmetry, we screened a variety of aromatic anions with matched symmetry and found an inhibitor, pentacyanocyclopentdienyl anion (PCCP^−^) that exhibited all characteristics of an open channel blocker. Inhibition was strongly dependent on the membrane potential. Through mutagenesis and covalent modification, we identified the region α_1_V256-α_1_T261 in the rat recombinant GABA_A_ receptor to be important for PCCP^−^ action. Introduction of positive charges into M2 increased the affinity for PCCP^−^ while PCCP^−^ prevented the access of a positively charged molecule into M2. Interestingly, other anion selective cys-loop receptors were also inhibited by PCCP^−^, among them the Drosophila RDL GABA_A_ receptor carrying an insecticide resistance mutation, suggesting that PCCP^−^ could serve as an insecticide.

## Introduction

Symmetry pervades nature at all levels from nuclear physics to astronomy [1]. In biology, it enables complex functions to arise from a limited set of building blocks and associated genes. A case in point is protein assemblies, such as viral capsids or trans-membrane ion channels. The former often show icosahedral symmetry, allowing for the encapsulation of maximum space with a minimum number of protein components [2]. The latter are often multimeric, for instance tetrameric (voltage-gated potassium channels), pentameric (cys-loop receptors) or hexameric (Orai channels), with a central pore formed by membrane-spanning subunits. Following the establishment of a basic multimeric assembly early in evolution, a higher level of functional sophistication is sometimes achieved through subsequent desymmetrization, for instance through concatenation or heteromultimerization of closely related, yet distinct, subunits.

**Figure 1:**
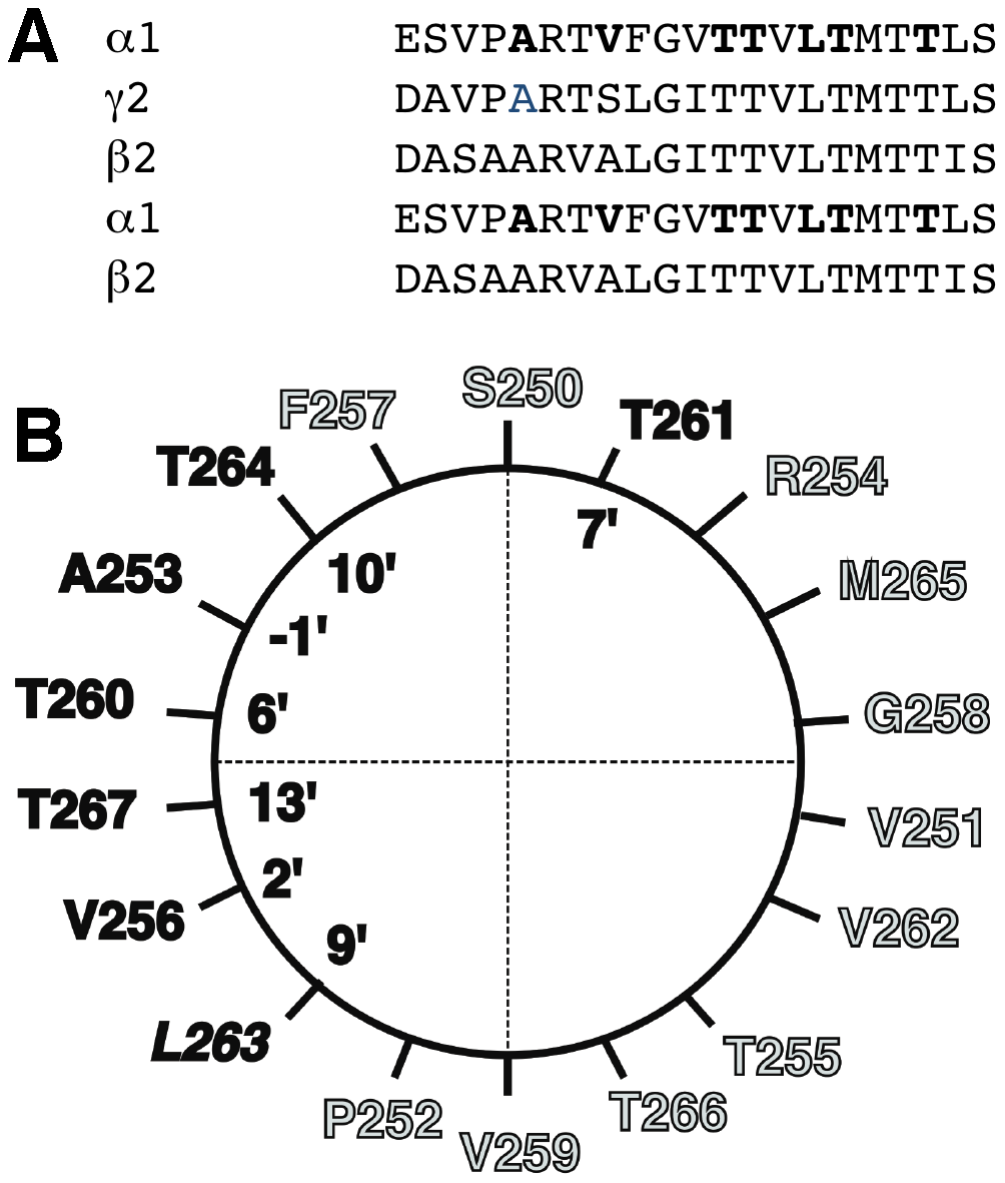
Aligned sequences of the amino acid residues in the subunits α_1_β_2_γ_2_ of the rat GABA_A_ receptor. A, Alignment of 2α, 2β and 1γ subunit contributing to the formation of a GABA_A_ pentamer. The residues in the α_1_ subunit of the GABA_A_ mutated to Cys are shown in boldface letters. B, α-Helical wheel representation of the rat α_1_ M2 membrane-spanning domain showing the mutated residues in boldface letters.

GABA_A_ receptors are a particularly interesting class of pentameric ligand-gated ion channels. They are composed of five subunits surrounding a central chloride ion channel and represent the major inhibitory receptors in the mammalian central nervous system [3]–[6]. The most abundant receptor isoform in mammalian brain consists of α_1_, β_2_, and γ_2_ subunits [7]. Various approaches have been used to derive the subunit stoichiometry for this receptor, which has been determined as 2α∶2β∶1γ. with a subunit arrangement gβαβα anti-clockwise as seen from the synaptic cleft [8]–[12]. The pharmacological properties depend on subunit composition [13] and arrangement [14]. The subunits of GABA_A_ receptors share a high degree of homology with other subunits of the same receptors, as well as subunits of other Cys-loop receptors. All these receptors have a near five-fold symmetry. The degree of symmetry is especially high in the second trans-membrane domain M2 that lines the ion channel (Fig 1A).

**Figure 2:**
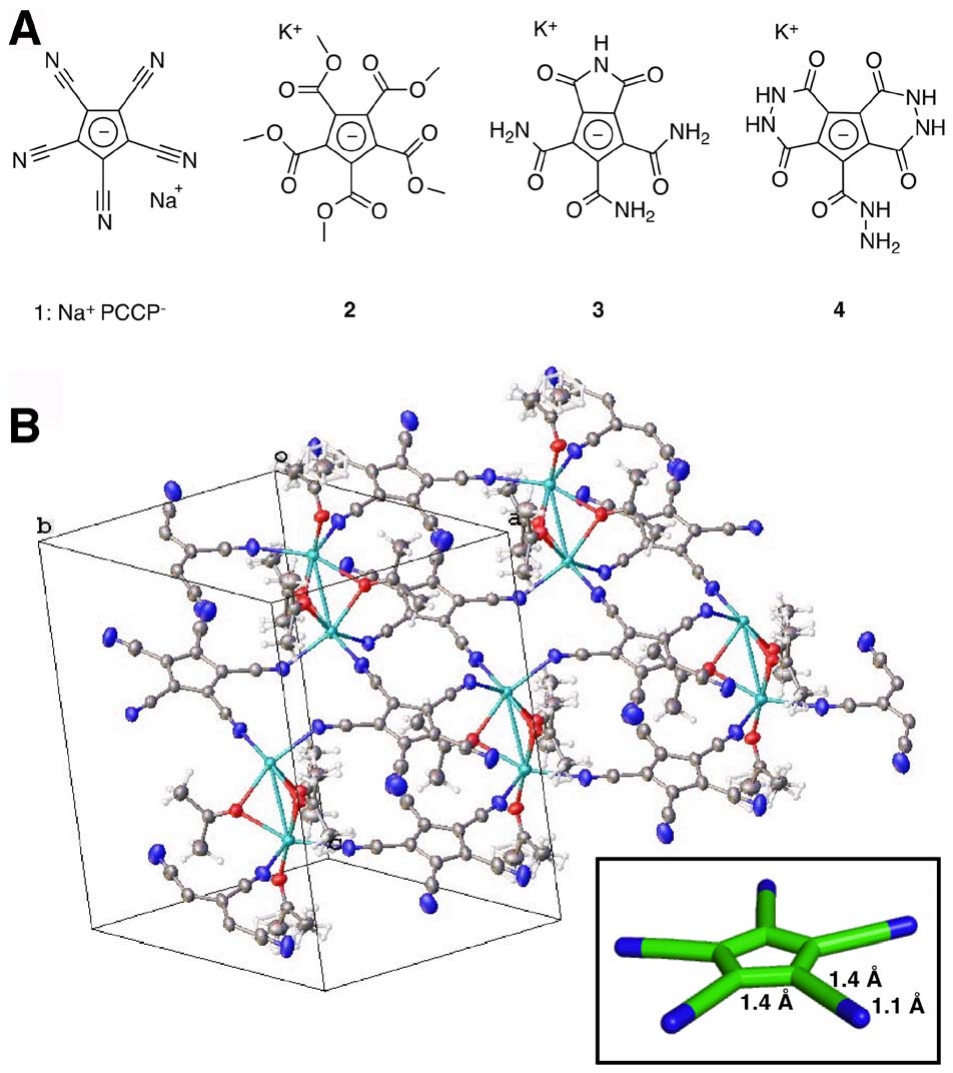
Symmetry-adapted anions, the chemical structure of PCCP^−^ and the X-ray structure of Na^+^PCCP^−^. A, Symmetry-adapted anions. B, X-ray structure of Na^+^PCCP^−^ (as the acetone solvate). The network of coordinative interactions between the partially negatively charged nitrogen atoms of PCCP^−^ and the Na^+^ cations is highlighted. The insert indicates the geometry of the molecule.

GABA_A_ receptors have a rich pharmacology and are targeted by numerous agents such as muscimol, picrotoxin, benzodiazepines and insecticides [15]. None of these ligands, however, takes advantage of the five-fold (or near five-fold) symmetry of the receptors and the availability of multiple, i.e. up to five, related contact sites. Encouraged by recent work on polyvalent ligands [16], we hypothesized that small pentasymmetric or nearly pentasymmetric anions would serve as symmetry-adapted blockers of the anion-selective GABA_A_ receptors. Such molecules would have multiple similar interactions with the protein, which would result in a sharp increase of overall binding affinity (avidity) due to the polyvalency effect [17]. To test this hypothesis, we synthesized a range of perfectly or nearly five-fold symmetric anions (Figure 2A) and investigated them in electrophysiological experiments. Among these, we identified the pentacyanocyclopentdienyl anion (PCCP^−^) as an inhibitor of GABA_A_ receptors. Here we describe that PCCP^−^ has all the hallmarks of an open channel blocker, discuss its binding site, and evaluate its interactions with other pentameric ligand-gated on channels.

## Materials and Methods

Compounds 1 (Na^+^PCCP^−^) and 2 were synthesized using established literature protocols. Compounds 3 and 4 were synthesized from 2 by treatment with ammonia and hydrazine, respectively. Details of these syntheses will be published elsewhere.

Crystallographic data (excluding structure factors) for Na^+^PCCP^−^ (acetone solvate) have been deposited with the Cambridge Crystallographic Data Centre as publication no. CCDC-946841. Copies of the data can be obtained free of charge on application to CCDC, 12 Union Road, Cambridge.

MTSET^+^ was obtained from Toronto Research Chemicals Inc. All the other chemicals were purchased from Sigma-Aldrich.

The recombinant rat mutant subunits α_1_A253C, α_1_V256C, α_1_T260C, α_1_T261C, α_1_L263C, α_1_T264C, α_1_T267C putatively facing the channel lumen (Fig. 1A,B) were prepared using the QuikChange mutagenesis kit (Stratagene).

Capped cRNAs were synthesized from the linearized plasmids. A poly-A tail of about 400 residues was added to each transcript using yeast poly-A polymerase. The concentration of the cRNA was quantified on a formaldehyde gel using Radiant Red stain for visualization of the RNA. Known concentrations of RNA ladder were loaded as standard on the same gel. cRNAs were precipitated in ethanol/isoamylalcohol 19∶1, the dried pellet dissolved in water and stored at −80°C. Xenopus oocytes were prepared, injected and defollicated as described previously [18] (Research approved by the Kantonstierarzt, Kantonaler Veterinärdienst Bern (Animal research permit BE98/12)). Briefly, Xenopus laevis oocytes were injected with 50 nL of the cRNA solution containing wild type or mutated α_1_, β_2_ and γ_2_ subunits at a concentration of 10 nM: 10 nM: 50 nM and then incubated in modified Barth's solution at 18°C for at least 24 h before the measurements. Homomeric glycine receptors (β-subunit), heteromeric glycine receptors (α and β-subunit) (cDNAs are a kind gift by B. Laube and H. Betz), the prokaryotic ELIC (cDNA is a kind gift by R. Dutzler), the wild-type and the dieldrin resistant (RDL) mutant Drosophila GABA_A_ receptor (wild type, bd splice variant and mutant A301S) (cDNAs are a kind gift by D. Sattelle) were also expressed. Currents were measured using a home-built two-electrode voltage clamp amplifier in combination with a XY-recorder or digitized using a PowerLab 2/20 (AD Instruments) using the computer program Chart. Tests with a model oocyte were performed to ensure linearity in the larger current range. The response was linear up to 15 µA. Electrophysiological experiments were performed by using the two-electrode voltage clamp method at a holding potential of −80 mV. The perfusion medium contained 90 mM NaCl, 1 mM KCl, 1 mM MgCl_2_, 1 mM CaCl_2_, and 5 mM Na-HEPES (pH 7.4) and was applied by a gravity flow of 6 ml/min. Wild type and and mutant receptors were characterized for their apparent affinity for γ-aminobutyric acid (GABA) for channel gating and for inhibition by PCCP^−^ and picrotoxin. The GABA concentration response curve was determined by sequential application of increasing concentrations of GABA. Concentration-inhibition curves were performed at GABA (EC_10_) by sequential co-application of GABA and increasing concentrations of PCCP^−^ or picrotoxin. Inhibition was determined at the end of 1 min co-application of GABA and PCCP^−^ or picrotoxin. Concentration response curves were fitted with I(c) =  I_max_/(1+(c/EC_50_)∧n, where I is the current potentiation, c is the concentration of GABA, I_max_ is the maximal current amplitude, EC_50_ is the concentration of GABA at which a half-maximal current amplitude was observed and n is the Hill coefficient. Concentration inhibition curves were fitted with I(c)  =  I_max_/(1+ (IC_50_/c)), where I is the control current amplitude, c is the concentration of PCCP^−^ or picrotoxin, I_max_ is the control current amplitude elicited by GABA and IC_50_ is the concentration of PCCP^−^ or picrotoxin at which half-maximal inhibition was observed. Drugs were applied as follows: 1 min GABA (EC_10_), 1 min GABA (EC_10_), 1 min GABA (EC_10_) + PCCP^−^ (IC_50_), 1 min MTSET^+^ (5 mM) either in the presence or absence of 100 µM GABA, 1 min GABA (EC_10_), 1 min GABA (EC_10_), 1 min GABA (EC_10_) + PCCP^−^ (concentration as before). Similar experiments were performed with picrotoxin. Due to the difficulty of washing out picrotoxin out from the oocytes two different oocytes were used to test the inhibition before and after the treatment with the cysteine reactive compound. Inhibition after treatment was divided by % inhibition before treatment. To test the ability of PCCP^−^ to protect the engineered cysteines from covalent modification by MTSET^+^, we used the same sequence of perfusion except that MTSET^+^ was co-applied with 1 mM PCCP^−^. Results were obtained on 3–4 single oocytes for each receptor.

The homology model is based on PDB entry 3RIF and was constructed with Modeller [19]. Ligand docking was performed with the GOLD software [20]. The binding site was defined to contain the 2′and 6′ residues, side chains α_1_T260, β_2_T256 and γ_2_T271 were kept flexible during docking.

## Results

### Synthesis and preliminary biological evaluation

Figure 2A shows the investigated symmetry-adapted anions. PCCP^−^ as its sodium salt [1] and compound 2 were synthesized following established literature procedures [21]–[23]. Compounds 3 and 4 were prepared from 2 by treatment with ammonia and hydrazine, respectively. Details of these syntheses will be published elsewhere. Compounds 1–4 were tested for inhibition of recombinant α_1_β_2_γ_2_ GABA_A_ receptors. Among these, only 1 was found to be highly active and was further characterized and the x-ray structure determined (Figure 2B).

### Low concentrations of PCCP^−^ inhibit currents mediated by α_1_β_2_γ_2_ GABA_A_ receptors

**Figure 3:**
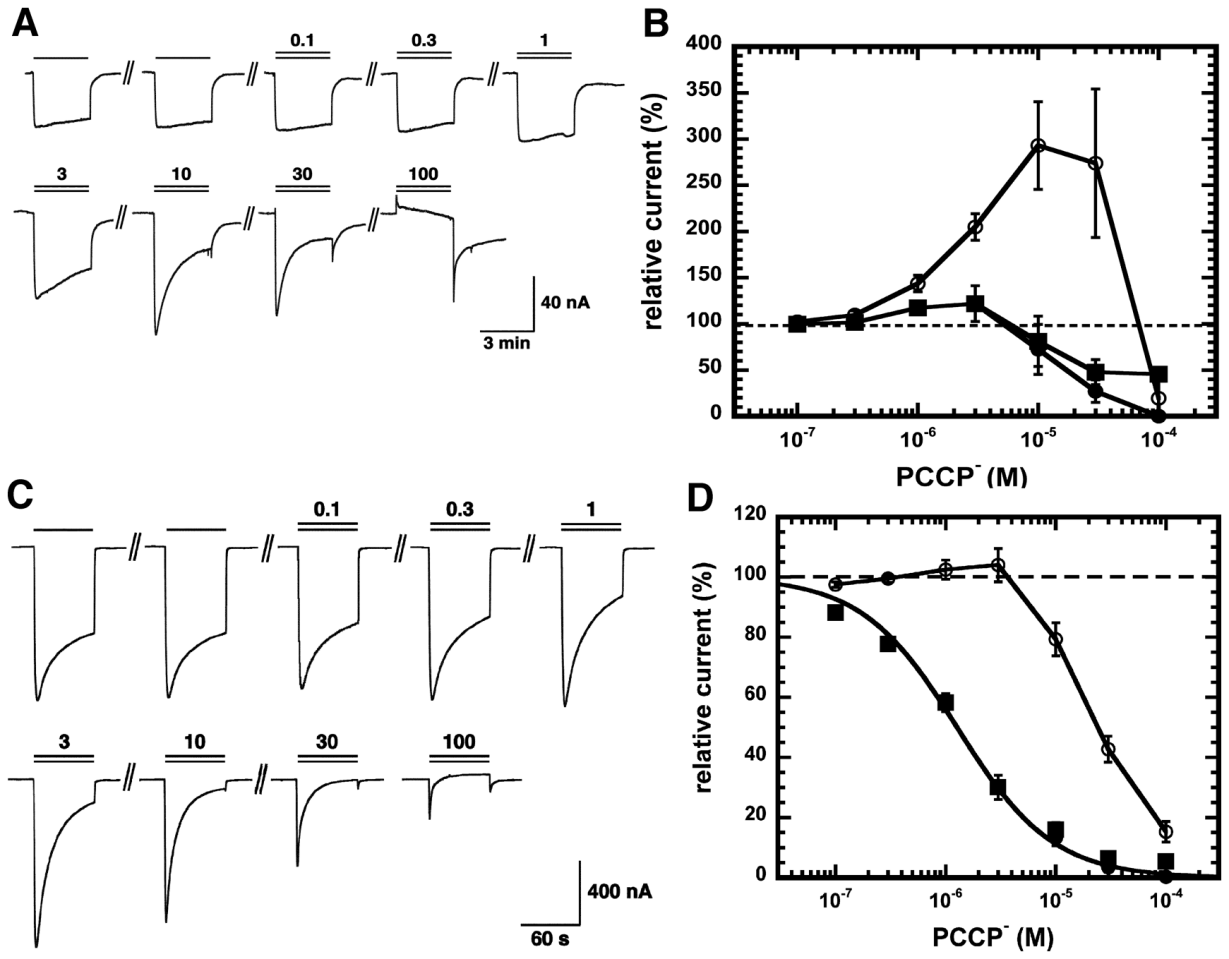
Effect of PCCP^−^ on recombinant α_1_β_2_γ_2_ GABA_A_ receptors. A, GABA_A_ receptors were expressed in Xenopus oocytes. The electrical currents recorded by two-electrode voltage clamp were activated with a concentration of GABA eliciting 1% of the maximal current amplitude (EC_1_) and inhibited with increasing concentrations of PCCP^−^. The lower bar indicates the time of GABA application, the upper bar the time of PCCP^−^ application. The numbers indicate the concentration of PCCP^−^ in µM. At concentrations >1 µM, induces an open-channel block, characterized by an apparent desensitization of the current and an off-current. B, Averaged concentration inhibition curve by PCCP^−^. Individual curves were fitted and standardized to the current elicited by GABA. Data are shown as mean ± SEM (n = 4). Open circle: peak current amplitudes at the beginning of the drug application. Filled squares: current amplitudes at the end of the drug application. Filled circles: current amplitudes at the end of the drug application corrected for the direct effect of PCCP^−^ on membranes. C) and D) same experiment carried out at a concentration of GABA eliciting 10% of the maximal current amplitude (EC_10_).

To evaluate PCCP^−^ as an ion channel blocker, α_1_β_2_γ_2_ rat GABA_A_ receptors were expressed in Xenopus oocytes. At concentrations of 0.3–10 µM PCCP^−^ stimulated currents elicited by low concentrations of GABA (EC_1_). This stimulation was highly variable between individual oocytes and was not mediated by the site for benzodiazepines as 1 µM Ro15-1788 fails to affect the stimulation. As stimulation was only observed at low concentration of GABA, we are tempted to assume a different site of action of PCCP^−^ for stimulation and inhibition. At higher concentrations (>1 µM), PCCP^−^ induced an open-channel block, characterized by an apparent desensitization of the current and an off-current. As expected, this block became more prominent with increasing agonist concentrations. Stimulation became less evident. Original current traces and averaged data are shown in Figure 3A,B.

Figure 3C,D shows a similar experiment carried out at a higher GABA concentration (EC_10_). At this GABA concentration, PCCP^−^ only exhibited a channel block. Current amplitudes measured after 1 min application of GABA and PCCP^−^ were fitted with an IC_50_ of 2.6±0.8 µM (n = 4). In additional experiments PCCP^−^ was pre-applied for 30 s before the combined application of PCCP^−^ with GABA. Current traces looked the same as without pre-application, indicating that PCCP^−^ did not interact with closed channels.

**Figure 4:**
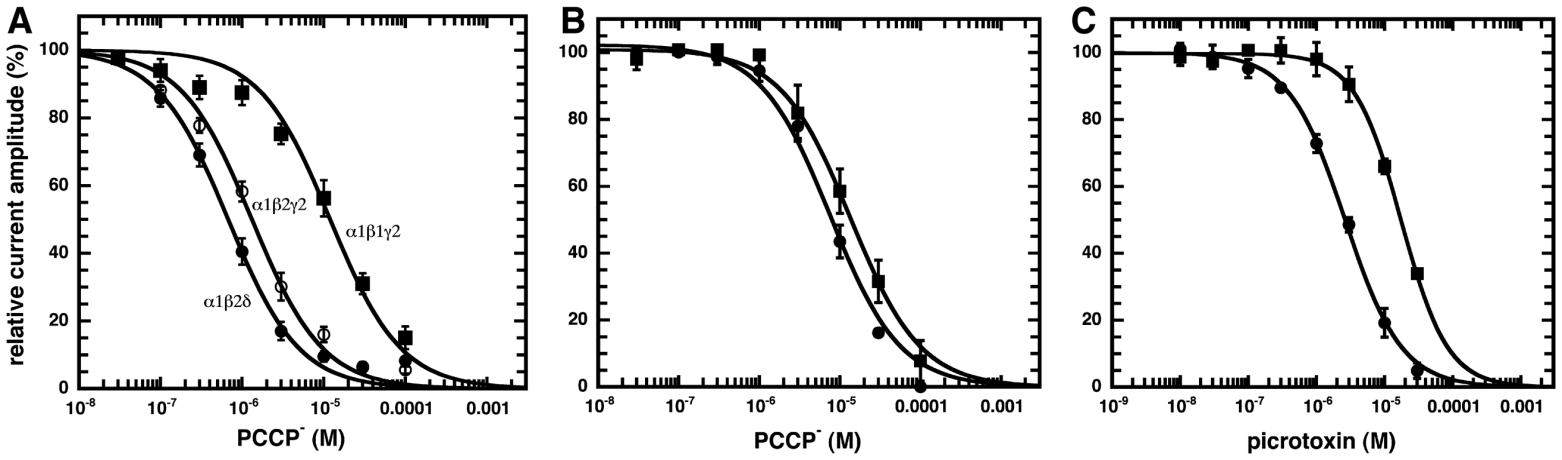
Concentration inhibition curves for rat and Drosophila GABA_A_ receptors. A, Concentration inhibition curve for α_1_β_2_γ_2_ and α_1_β_2_δ GABA_A_ receptors. Increasing concentrations of PCCP^−^ were applied together with GABA EC_10_. Individual inhibition curves were standardized and subsequently averaged (Mean ± SD, n = 4). Data are compared with those obtained from α_1_β_2_γ_2_ receptors. Inhibition of the wild type and mutant RDL Drosophila GABA_A_ receptor by PCCP^−^ and Picrotoxin. Concentration inhibition curves of (B) PCCP^−^ and, (C) picrotoxin were determined in wild type (circles) and mutant (squares) receptors. Currents were activated with a concentration of GABA eliciting 10% of the maximal current amplitude (EC_10_) and inhibited with increasing concentration of PCCP^−^ or picrotoxin. Individual curves were standardized to initial current amplitudes and subsequently averaged. Data are shown as mean ± SD (n = 3).

PCCP^−^ also inhibited α_1_β_2_γ_2_ and α_1_β_2_δ rat GABA_A_ receptors, respectively, with an IC_50_ of 12.5±4.8 µM (n = 4) and 0.71±0.28 µM (n = 4) (Figure 4A). It should be noted that the primary sequences of β_1_ and β_2_ differ substantially in the inner leaflet of M3. Glycine homomeric and heteromeric receptors [24] were similarly inhibited, while ELIC [25] required about 100 µM PCCP^−^ for half-maximal inhibition.

**Figure 5:**
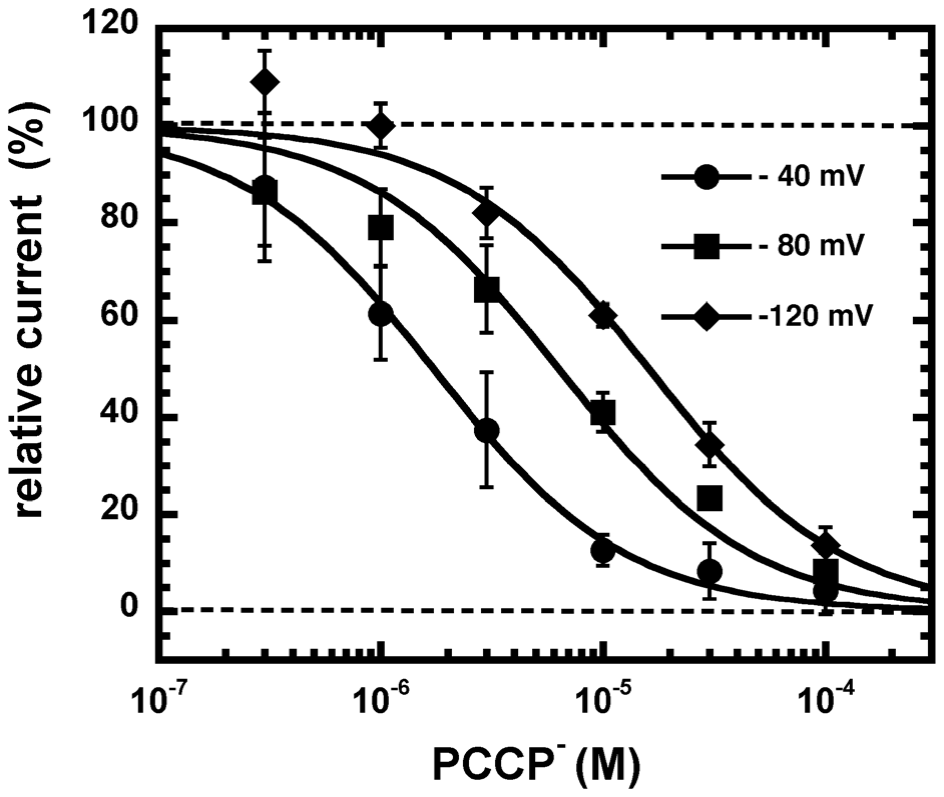
Effect of the membrane potential on inhibition by PCCP^−^. A, GABA_A_ receptors were activated with a concentration of GABA eliciting 10% of the maximal current amplitude (EC_10_) and inhibited with increasing concentrations of PCCP^−^. Averaged concentration inhibition curves by PCCP^−^ are shown for different membrane potentials. Individual curves were fitted and standardized to the current elicited by GABA. Data are shown as mean ± SD (n = 3).

Inhibition by PCCP^−^ was strongly dependent on the membrane potential (Figure 5). IC_50_ was 16.2±1.3 µM (n = 3) at −120 mV, 6.3±1.3 µM (n = 3) at −80 mV and 1.8±0.8 µM (n = 3) at −40 mV. It should be noted that the IC_50_ at −80 mV was for unknown reasons somewhat higher than determined in the experiments before. From these values one can estimate the fraction of the voltage field experienced by the blocking particle at its blocking site from the equation derived by Woodhull [26] where δ is the fraction of the voltage field sensed by the blocker from the outside of the membrane. δ was estimated to be ∼0.7 the distance of the voltage field from the extracellular side.

In additional experiments it was tested of inhibition by PCCP^−^ was of competitive or non-competitive nature. GABA concentration response curves were carried out in the absence of PCCP^−^ or the presence of 2 µM or 10 µM PCCP^−^. Analysis was complicated by the fact that currents were determined after 1 min application of GABA, when a substantial proportion of the channels had been desensitized. Nevertheless data showed rather a leftward shift of the GABA concentration response curve with increasing concentrations of PCCP^−^, a phenomenon excluding competitive inhibition.

### High concentrations of PCCP^−^ interact with the lipid bilayer

**Figure 6:**
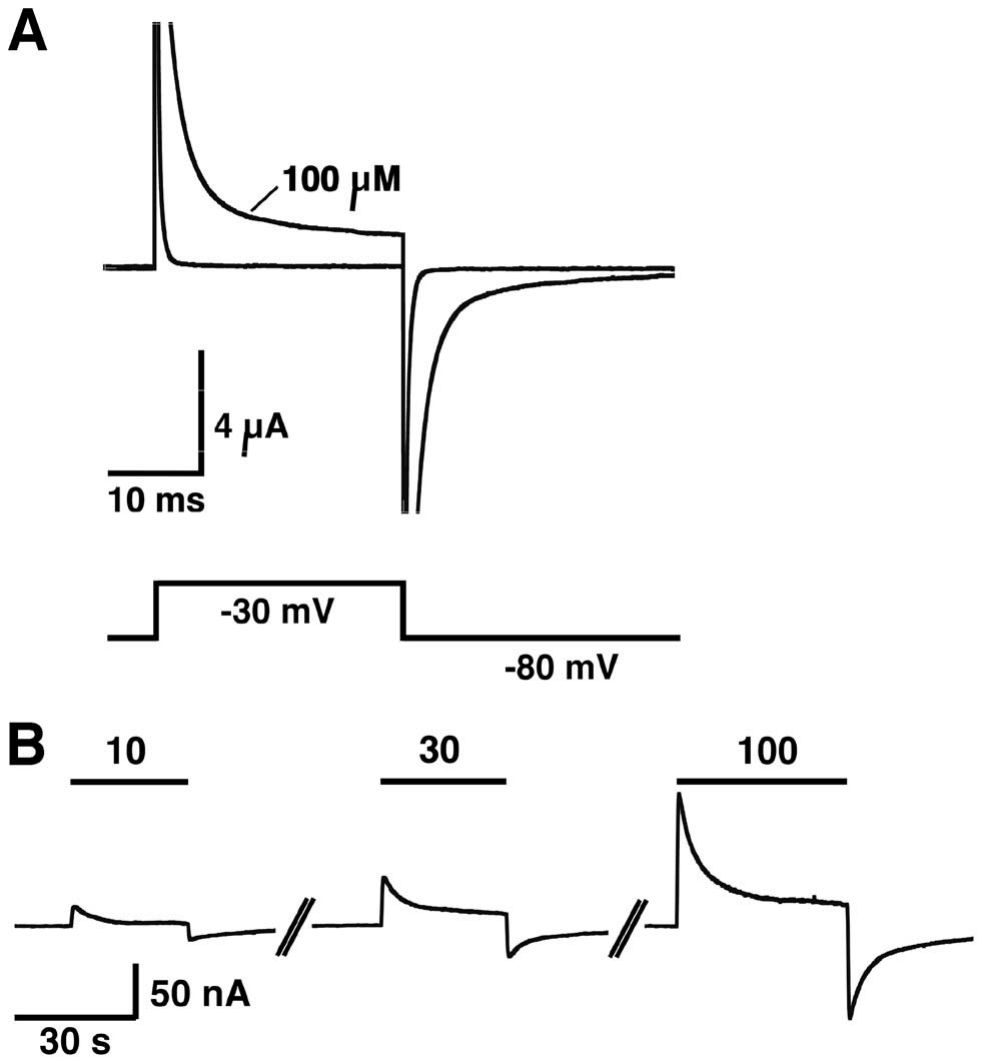
High concentration of PCCP^−^ induce a current in non-injected Xenopus oocytes. A, Voltage jump of 25 ms duration from −80 mV to −30 mV. A µA sized transient current flows with each voltage step. B, A small transient outward current is induced after applications of 10, 30 and 100 µM PCCP^−^ of 30 s duration to an oocyte held at a membrane potential of −80 mV.

At concentrations ≥10 µM, PCCP^−^ induced a current in non-injected oocytes. Perfusion of 10, 30 or 100 µM PCCP^−^ at a holding potential of −80 mV resulted in small outward currents (Figure 6B). Upon a voltage jump from −80 to −30 mV, µA sized currents of more than 30 ms duration were observed in the presence of 100 µM PCCP^−^ (Fig. 6A). Thus it appears that PCCP^−^ is able to insert into the bilayer and diffuse through the bilayer, reflecting its lack of dipole moment and relative lipophilicity (clogP  = −0.48). It should be noted that the concentrations of PCCP^−^ required to induce currents in non-injected oocytes are much larger than the concentrations required to inhibit GABA_A_ receptor channels.

### PCCP^−^ inhibits currents mediated by wild type and mutant RDL Drosophila GABA_A_ receptor to a similar extent

Mutation A302S in RDL Drosophila GABA_A_ receptors has been reported to confer a certain degree of resistance to inhibition by picrotoxin [27], [28]. In our hands, a 7-fold reduction in sensitivity to picrotoxin upon the mutation was observed. Interestingly, PCCP^−^ showed a much smaller (about 2-fold) difference in the IC_50_ between wild type and mutant receptors (Figure 4B.C).

### Pharmacological properties of mutant receptors

**Figure 7:**
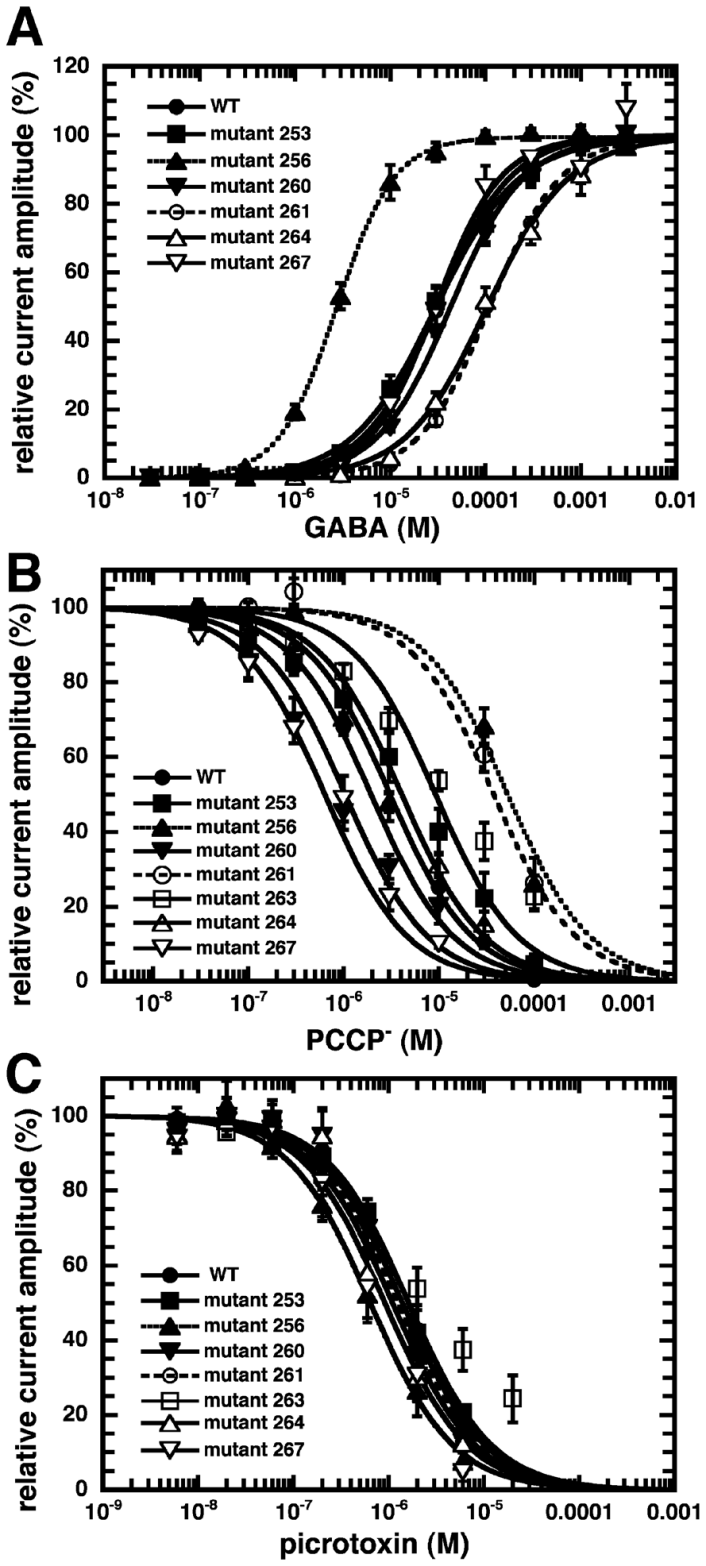
Pharmacological properties of wild type and cysteine mutant GABA_A_ receptors. A, GABA concentration response curves from wild type and mutant rat GABA_A_ receptor. B, PCCP^−^ and C, picrotoxin concentration inhibition curves. Individual curves were fitted and standardized. Data are shown as mean ± SD (n = 3 to 4).

To identify important residues for the interaction with PCCP^−^, we mutated, one at a time, seven residues in M2 of α_1_ to cysteine residues. (Figure 1A,B). Mutated α_1_ subunits were co-expressed with wild type β_2_ and γ_2_ subunits in Xenopus oocytes. The sensitivity to GABA was tested for the different mutants. Expression of α_1_L263Cβ_2_γ_2_ resulted in a spontaneously open channel. While α_1_A253Cβ_2_γ_2_, α_1_T260Cβ_2_γ_2_, α_1_T267Cβ_2_γ_2_ showed an EC_50_ similar to wild type receptors, the mutant α_1_V256Cβ_2_γ_2_ showed an about 10-fold increase and the mutants α_1_T261Cβ_2_γ_2_ and α_1_T264Cβ_2_γ_2_ showed an about 4 fold decrease in the EC_50_ for GABA (Figure 7A; Table 1). The sensitivity to inhibition by PCCP^−^ of GABA-activated currents was also determined for wild type and mutant receptors. A GABA concentration eliciting 10% of the maximal current in the corresponding receptor was used in these experiments. The mutant receptors α_1_V256Cβ_2_γ_2_ and α_1_T261Cβ_2_γ_2_ displayed each an about 20–30 fold reduced sensitivity to PCCP^−^ compared with wild type receptors (Figure 7B; Table 1). The sensitivity of GABA-activated currents to picrotoxin was also determined using the same conditions as for PCCP^−^ (Fig. 7C; Table 1). Little effect of the studied mutations was observed on the IC_50_ for picrotoxin.

**Table 1:** **Pharmacological evaluation of the expressed recombinant receptors.**

Receptor	GABA EC_50_ (µM) mean ± SD	n	PCCP^−^ IC_50_ (µM) mean ± SD	n	Picrotoxin IC_50_ (µM) mean ± SD	n
α_1_β_2_γ_2_	31.3±6.5	3	2.58±0.75	3	2.02±0.89	6
α_1_A253Cβ_2_γ_2_	30.6±6.4	3	2.63±0.31	3	1.79±0.15	3
α_1_V256Cβ_2_γ_2_	2.91±7.4	4	62.2±22.4	4	0.74±0.36	4
α_1_T260Cβ_2_γ_2_	44.1±6.4	4	0.85±0.46	4	1.31±0.41	3
α_1_T261Cβ_2_γ_2_	111±1	3	46.4±7.25	3	1.31±0.71	3
α_1_L263Cβ_2_γ_2_	open ch.	3	4.47±1.36	3	1.10±0.21	3
α_1_T264Cβ_2_γ_2_	126±24	4	3.15±0.48	3	1.03±0.35	3
α_1_T267Cβ_2_γ_2_	32.2±11.9	3	0.77±0.11	3	0.83±0.48	3

EC_50_ for GABA, IC_50_ for PCCP^−^, and IC_50_ for picrotoxin are given for wild type and mutant receptors.

### The effect of MTSET^+^ on the cysteine-substitution mutants and the binding site of PCCP^−^

**Figure 8:**
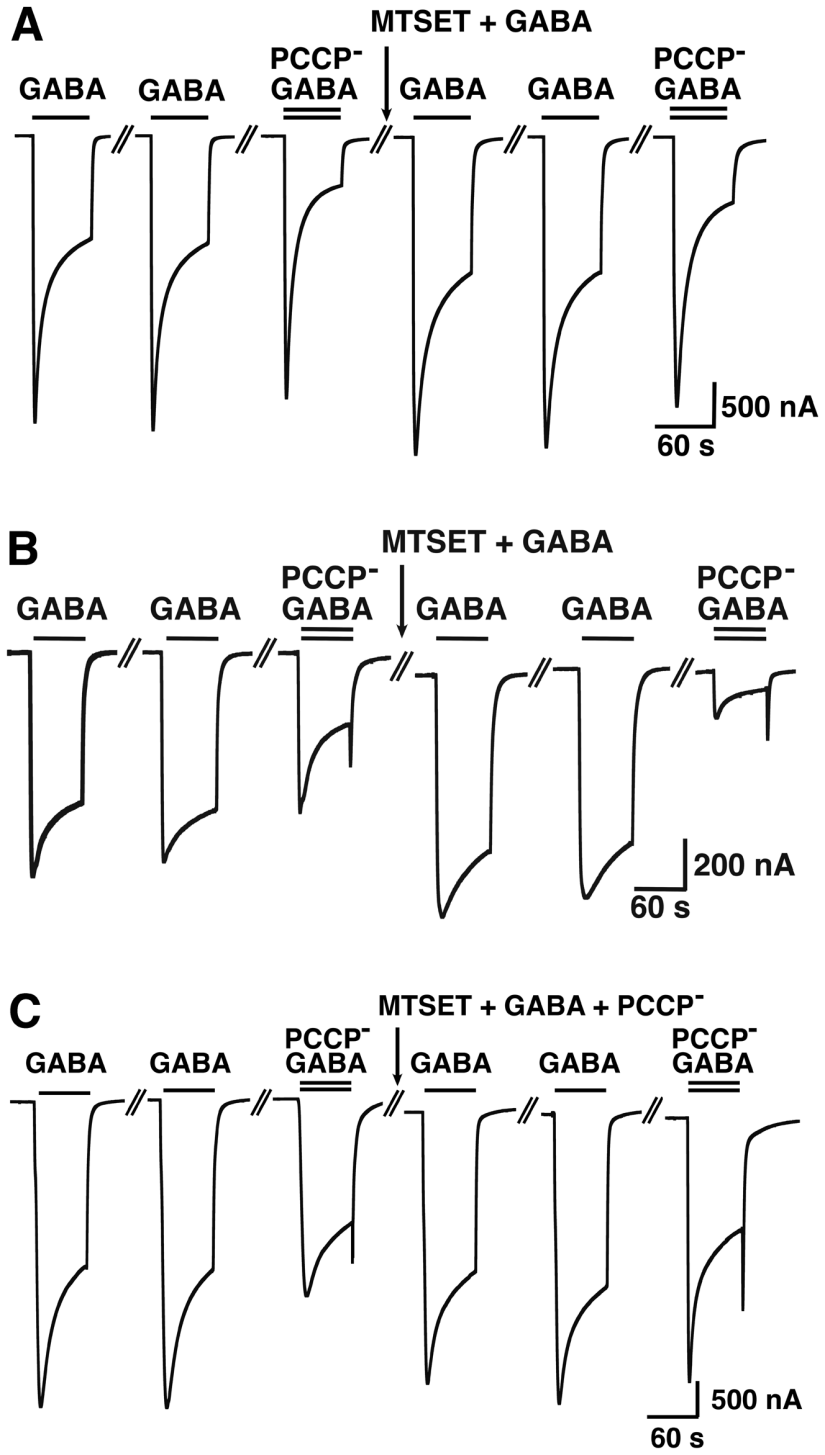
PCCP^−^ prevents the increase in PCCP^−^ sensitivity of α_1_V256β_2_γ_2_ mediated by MTSET^+^ + GABA. GABA (EC_10_) was applied repetitively until a stable current response was observed followed by inhibition of the channel by PCCP^−^. Subsequently 5 mM MTSET was applied in the presence of GABA. After MTSET^+^ treatment GABA was applied twice followed by a combined application of GABA and the same concentration of PCCP^−^ used before. A, Wild type receptors were not affected by this treatment. B, The treatment leads to an enhanced inhibition in α_1_V256Cβ_2_γ_2_. C, 5 mM MTSET^+^ was applied to α_1_V256C mutant receptor in presence of GABA and 1 mM PCCP^−^. PCCP^−^ prevented enhanced inhibition and therefore covalent reaction. These experiments were repeated independently three times using different oocytes.

**Figure 9:**
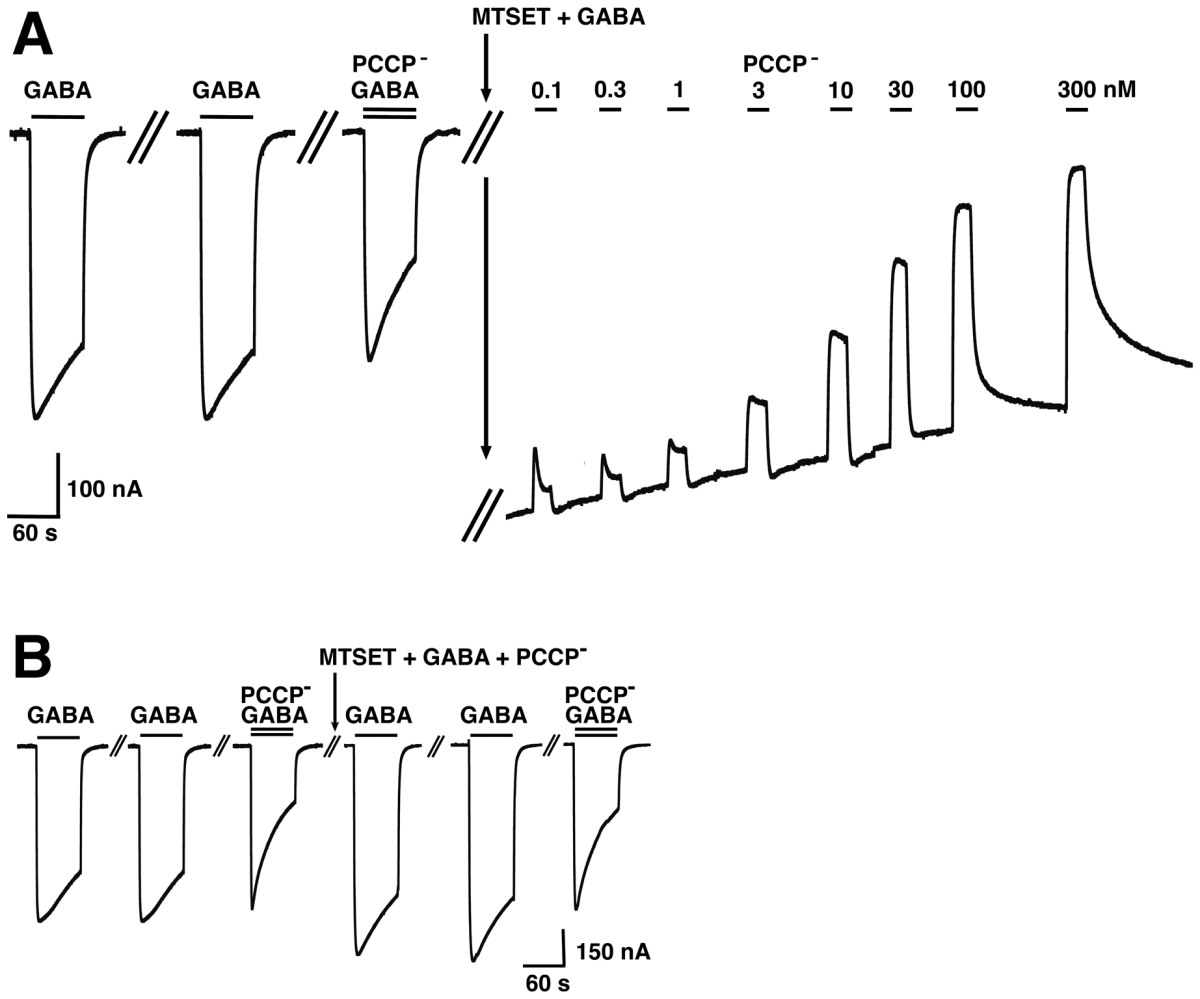
PCCP^−^ prevents the increase in PCCP^−^ sensitivity of α_1_T260β_2_γ_2_ mediated by MTSET^+^ + GABA. GABA (EC_10_) was applied repetitively until a stable current response was observed followed by inhibition of the channel by PCCP^−^. Subsequently 5 mM MTSET^+^ was applied in the presence of GABA. After MTSET^+^ treatment GABA was applied twice followed by a combined application of GABA and the same concentration of PCCP^−^ used before. A, The treatment leads to a spontaneously open channel and an enhanced inhibition in α_1_T260Cβ_2_γ_2_. B, 5 mM MTSET^+^ was applied to α_1_T260C mutant receptor in presence of GABA and 1 mM PCCP^−^. PCCP^−^ prevents the open channel formation. These experiments were repeated independently three times using different oocytes.

**Figure 10:**
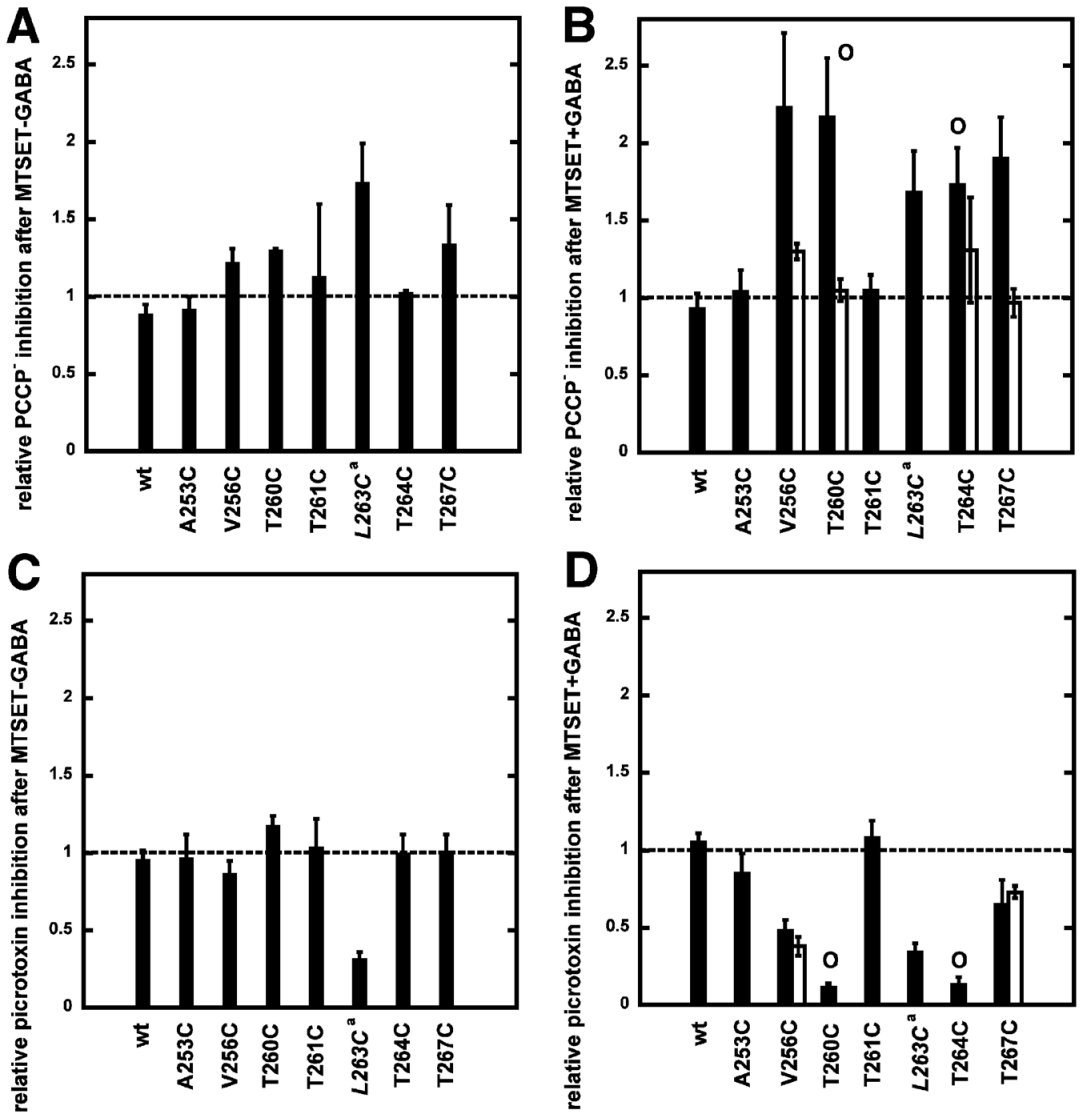
Effect of MTSET^+^ on the PCCP^−^ and picrotoxin inhibition of wild type and mutants GABA_A_ receptors. Currents were elicited with GABA EC_10_. The concentration of PCCP^−^ or picrotoxin was chosen such as to inhibit about 50% of the late current response in the corresponding receptor. Subsequently oocytes were treated with the cysteine-reactive reagent MTSET^+^ and inhibition by the same concentration of PCCP^−^ or picrotoxin was determined. The ratio of the inhibition after treatment divided by inhibition before treatment is shown as a bar. A, C, MTSET^+^ was applied in the absence of GABA. B, D, MTSET^+^ was applied in the presence of 100 µM GABA. The circle symbol on top of the bar for the T260C and T264C mutations indicates formation of an open channel after MTSET^+^ treatment when applied in the presence of GABA. The white bars show results of experiments where the MTSET^+^ + GABA treatment was performed in the presence of 1 mM PCCP^−^ or 1 mM picrotoxin, in order to see if the covalent reaction could be prevented by the channel blockers. The asterisks sign (*) indicates that 1 mM picrotoxin was not able to suppress formation of open channels. Mean ± SD is shown. The number of oocytes for each experimental condition is either three or six.

A cysteine scan was chosen as it allows covalent reaction with a cysteine reactive compound to potentially increase the effect seen with the mutation alone, to test accessibility of the residue and to investigate protection from the covalent reaction by a compound. In preliminary experiments pCMBS^−^, MTSEA^+^ and MTSET^+^ were tested. Only treatment with MTSET^+^ left wild type GABA_A_ receptors unaffected (Figure 8A). Therefore MTSET^+^ was chosen for further experimentation. Current traces for wild type receptor and α_1_V256Cβ_2_γ_2_ and α_1_T260Cβ_2_γ_2_ mutant receptors are shown in Figure 8B,C and Figure 9A,B, illustrating typical experiments where we chose an inhibitor concentration such as to inhibit about 50% of the late current response elicited by GABA (EC_10_) in the corresponding receptor. MTSET^+^ + GABA treatment applied for 1 min to the wild type receptor had no effect on the affinity for PCCP^−^ (Figure 8A). MTSET^+^ + GABA treatment in α_1_V256Cβ_2_γ_2_ led to an increase in PCCP^−^ inhibition (Figure 8B). This effect could be prevented when 1 mM of PCCP^−^ was present during the treatment (Figure 8C). The Figure 9A shows that MTSET^+^ + GABA treatment leads to a spontaneously open channel in the α_1_T260Cβ_2_γ_2_ mutant, characterized by a shift on the base line of the current. We applied increasing concentrations of PCCP^−^ to investigate if PCCP^−^ was able to block the channel again. The IC_50_ value after the treatment was 0.90±0.3 nM (mean ± SD, n = 3) as compared to the IC_50_ of 0.85±0.46 µM (n = 3) before the treatment. This indicates a strongly enhanced affinity for PCCP^−^. Moreover, as the maximally inhibited current level almost reached the original base line we conclude that PCCP^−^ was able to close the channel again. Figure 9B documents that presence of 1 mM PCCP^−^ during MTSET^+^ + GABA treatment prevents the open channel formation and the affinity for PCCP^−^ was the same before and after the treatment. Figure 10 summarizes our observations in wild type and mutant receptors. The bars indicate the ratio of the percentage of inhibition by PCCP^−^ (or picrotoxin) observed after application divided by that before the application of MTSET^+^ (black bars). The diagrams on the left show this ratio for PCCP^−^ (Figure 10A) and picrotoxin (Figure 10C) after application of MTSET^+^ without GABA and the diagrams on the right show the above ratio for PCCP^−^ (Figure 10B) and picrotoxin (Figure 10D) after application of MTSET^+^ in the presence of GABA. A value of 1 indicates that the IC_50_ before and after MTSET^+^ treatment were the same. An increase in the value indicates a decrease in the IC_50_ of an inhibitor (increase in apparent affinity) and a decrease below 1 the opposite. MTSET^+^ treatment in the absence of GABA caused generally little change in the apparent affinity of both channel inhibitors (Figure 10A,C). An exception is the mutant α_1_L263Cβ_2_γ_2_ with a significant increase in the ratio for PCCP^−^ (p<0.01) and a significant decrease in the ratio for picrotoxin (p<0.001). This mutation leads to an open channel, to the lumen of which MTSET^+^ obviously has access in the absence of GABA. For those mutations already slightly affected by the MTSET^+^ treatment alone, the effect on the inhibition by PCCP^−^ increased when the MTSET^+^ treatment was carried out in presence of 100 µM GABA. The application of MTSET^+^ + GABA caused a significant increase in the % inhibition by PCCP^−^ in α_1_V256Cβ_2_γ_2_, α_1_T260Cβ_2_γ_2_, α_1_T263Cβ_2_γ_2_, α_1_T264Cβ_2_γ_2_ and α_1_T267Cβ_2_γ_2_ (each p<0.01) mutants (Figure 9B). Upon co-application of MTSET^+^ with GABA, covalent reactions resulted in the formation of an open channel in α_1_T260Cβ_2_γ_2_ and α_1_T264Cβ_2_γ_2_. These open channel currents could not be further enhanced by GABA. The application of MTSET^+^ + GABA also caused a significant decrease in the % inhibition by picrotoxin in α_1_V256Cβ_2_γ_2_, α_1_T260Cβ_2_γ_2_, α_1_T264Cβ_2_γ_2_ and α_1_T267Cβ_2_γ_2_ (each p<0.005) mutants (Figure 10D). To investigate if the covalent reaction after MTSET^+^ + GABA treatment could be prevented by the channel inhibitors, we co-applied 1 mM PCCP^−^ or picrotoxin during MTSET^+^ + GABA treatment. In this case the ratio between the inhibition by PCCP^−^ or picrotoxin after treatment, divided by that before the treatment is represented by the white bars. The increase in PCCP^−^ sensitivity in the studied mutant receptors could be prevented in the presence of 1 mM PCCP^−^ during MTSET^+^ + GABA application for all the mutants affected by the cysteine reactive compound (Figure 10B). The decrease in picrotoxin sensitivity could not be prevented in the presence of 1 mM picrotoxin during MTSET^+^ + GABA application (Figure 10D).

## Discussion

Based on symmetry considerations, we have identified an aromatic monovalent anion with five-fold symmetry, PCCP^−^, as inhibitor of rat GABA_A_ receptors. The exposure to increasing concentrations of PCCP^−^ causes inhibition in the GABA-evoked current typical for an open channel blocker. All the pentameric receptors belonging to the Cys-loop family share a near five-fold symmetry which is most pronounced in the second transmembrane domain M2. PCCP^−^ can also inhibit glycine homomeric and heteromeric receptors with similar affinity to the GABA_A_ receptor and with smaller affinity ELIC. Interestingly, PCCP^−^ also inhibits Drosophila wild type and mutant RDL channels carrying the dieldrin resistance mutation [27], [28] suggesting a possible use of PCCP^−^ as insecticide. High concentrations of PCCP^−^ induced currents by themselves. We ascribe this phenomenon to distribution of PCCP^−^ into the lipid bilayer and permeation through the bilayer. PCCP^−^ lacks a dipole moment and is therefore comparatively lipophilic with a clogP of −0.48. However, we can not exclude an action of PCCP^−^ on channels endogenous to the oocyte.

We studied the molecular site of interaction of PCCP^−^ on α_1_β_2_γ_2_ GABA_A_ receptors. A series of cysteine mutations were introduced in M2 into amino acid residues of the α_1_ subunit. We selected residues that have been proposed to line the ion channel α_1_A253, α_1_V256, α_1_T260, α_1_T261, α_1_L263, α_1_T267 [29], [30] (Fig. 1A,B). The mutant receptors α_1_L263Cβ_2_γ_2_ form an open channel that could not be activated by GABA. This leucine residue is conserved in all known subunits of acetylcholine, glycine and GABA_A_ receptors. It has been postulated that this residue plays a role in the gating mechanism of the channel, where the closure is achieved when the large hydrophobic leucine residues move into the channel inhibiting ion flux (for review see [31]). Most of the mutations studied here had little effect on the apparent affinity to GABA. Mutations in residues α_1_V256 and α_1_T261 each caused an approximately 30–20 fold decrease in the apparent affinity of PCCP^−^ to inhibit currents induced by GABA. This may suggest that PCCP^−^ directly interacts with these residues, but it cannot be excluded here that these two residues allosterically affect the PCCP^−^ binding site. The mutations did not affect the apparent affinity for picrotoxin to inhibit current elicited by GABA.

It was of interest to test accessibility of the introduced cysteines to a cysteine reactive reagent. MTSET^+^ was chosen as it had a negligible effect on wild type receptors. It should be noted that a receptor pentamer has two α_1_ subunits and that covalent reaction of a mutated receptor with MTSET^+^ introduces two positive charges. Evidence was obtained that MTSET^+^ has better access to the channel lumen in the presence of the channel agonist GABA and can penetrate as far as α_1_V256. Similar observations have been made by Xu et al. [32] using a different cysteine reactive reagent.

**Figure 11:**
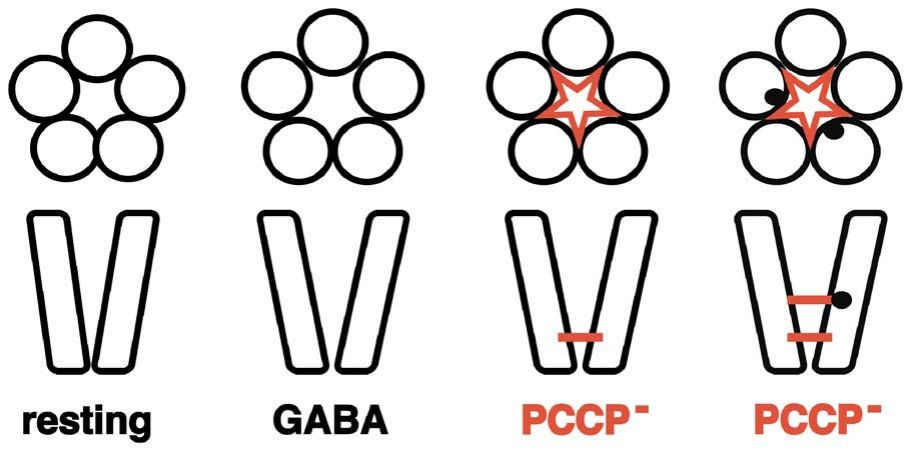
Hypothetical model for the mechanism of action of PCCP^−^. Mutation of residues α_1_V256 and α_1_T261 to cysteine alters strongly the apparent affinity for channel inhibition by PCCP^−^. The fact that MTSET^+^ can only react with cysteines introduced in M2 in the presence of GABA indicates that GABA widens the pore. For the mutations α_1_V256C and α_1_T260C the affinity for PCCP^−^ is strongly increased after MTSET^+^ treatment. MTSET^+^ reaction is prevented by PCCP^−^. This together implies these residues in PCCP^−^ binding. Introduction of two positive charges by reaction with MTSET^+^ further up in the channel leads to additional binding sites for PCCP^−^.

Interestingly, covalent reaction of MTSET^+^ with α_1_V256Cβ_2_γ_2_, α_1_T260Cβ_2_γ_2_, α_1_L263Cβ_2_γ_2_, α_1_T264Cβ_2_γ_2_ and α_1_T267Cβ_2_γ_2_, led to an increase in the apparent affinity for PCCP^−^ for channel inhibition. The fact that introduction of a relatively bulky moiety leads to an increase in affinity is probably due introduction of positive charges that favorably interact with the negatively charged PCCP^−^. Covalent reaction of MTSET^+^ with α_1_V256Cβ_2_γ_2_, α_1_T260Cβ_2_γ_2_ and α_1_T267Cβ_2_γ_2_ was prevented in the presence of PCCP^−^. The simplest interpretation of our observations including the direct effect of the mutations α_1_V256C and α_1_T261C on the affinity of PCCP^−^ is that PCCP^−^ can penetrate almost down to the level of α_1_V256C. Binding of PCCP^−^ then prevents MTSET^+^ access by channel constriction (Figure 11). However, we cannot exclude that PCCP^−^ has a second binding site at the level of α_1_T267 that is not sensitive to the mutation of this residue to cysteine.

Hydrophobic anions have previously been described to inhibit GABA_A_ receptors in a voltage independent fashion [33] reportedly in the absence of a conventional ligand binding site. Similar to the observations made here the mutation of residue α_1_V256 located in M2 affected inhibition. We can not fully exclude the possibility of an action of PCCP^−^ outside the channel, but following observations are in line with the existence of a binding site for PCCP^−^ within the channel: a) off currents upon removal of PCCP^−^, b) site of action in the inner leaflet of the membrane (coinciding with the location of α_1_V256/α_1_T260/α_1_T261, c) increase in the affinity for PCCP^−^ after introduction of positive charges in the form of MTSEA^+^ into M2 and d) prevention by PCCP^−^ of the reaction of MTSEA^+^ in different positions.

**Figure 12:**
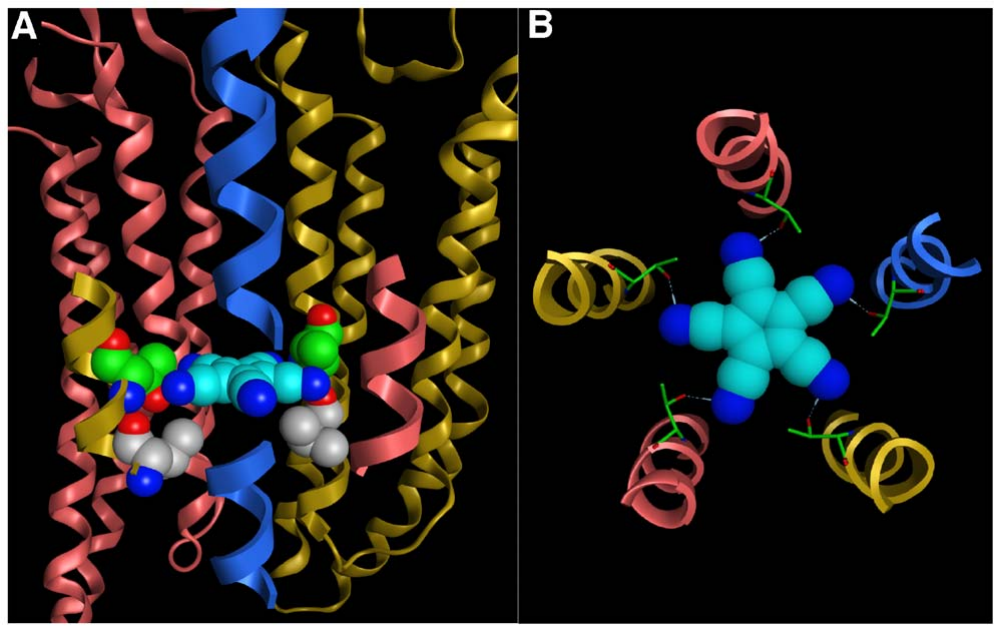
Molecular model of the interaction of PCCP^−^ with GABA_A_ receptors. A, The side view of the PCCP^−^ docking pose from the perspective of the γ_2_ subunit. The ligand and the mutated residues of the α_1_ subunit, which have an impact on the affinity of the ligand, are shown in space filling representation. The 2′ valines of the α_1_ subunit are rendered grey; the 6′ threonines of the α_1_ subunit in green. The GABA_A_ receptor is displayed in ribbon representation with α_1_ subunits shown in yellow, β_2_ subunits in red, γ_2_ subunit in blue. The complete transmembrane domain (TMD) is shown only of the α_1_ and the β_2_ subunits in the back. Of the subunits in front, only a segment of the transmembrane domain 2 (TMD2) is depicted. The TMD2 of the γ_2_ subunit is only partly displayed to provide a “window” through which the ligand is seen. B, Top view of the pose showing the symmetric molecular interactions between ligand and receptor. PCCP^−^ (space filling) forms H-bonds (blue dashed lines) to the –OH groups of the 6′ threonines (stick representation) of each of the five subunits.

PCCP^−^ is a rigid symmetric molecule with a diameter of approximately 10 Å that can engage in interactions with metals and form hydrogen bonds with its five peripheral nitrogen atoms. These bonds can extend along the C,N-axis or at a slightly bent angle (Fig. 1B). We think that the most likely interpretation of our findings is that PCCP^−^ blocks the receptor by plugging the pore at the level of α_1_V256C, α_1_T260 and α_1_T261, adopting a position parallel to the lipid bilayer. Figure 12A,B depicts a molecular model of the binding site of PCCP^−^. The ligand is in a planar position between the highly conserved 6′ level threonines (α_1_T260) forming H-bonds with the hydroxy groups, and the variable 2′ level of α_1_V256 and the homologous β_2_A251 and γ_2_T267. These protein-ligand contacts are consistent with the observed affinity differences for different pentamers, as the shape complementarity and surface properties of the 2′ level will be unique for each homo- or hetero-pentameric receptor. The relatively slow rate of block could be due to the strong tendency of PCCP^−^ to form H-bridges. It may be hypothesized that on the way down the channel lumen it interacts several times with the receptor.

It is interesting to compare the binding site for PCCP^−^ to that of picrotoxin, a well-known open channel blocker. Considerably controversy exists if picrotoxin occludes the channel directly or whether it allosterically affects the channel (reviewed in [34] and references therein). Possibly the best evidence for channel occupancy comes from crystallization experiments. The crystal structure of the homo-pentameric Caenorhabditis elegans glutamate-gated chloride channel α (GluCl) shows that picrotoxin (9 Å diameter) directly occludes the pore near its cytosolic base at the 2′ Thr and -2′ Pro side chains [35]. In this position, the channel diameter in static condition of the crystal is 4.6 Å. These residues are homologous to P252 and V256 in the M2 of the α_1_ GABA_A_ receptor subunit. Thus, it appears that PCCP^−^ and picrotoxin occupy sites in the lumen of the channel toward the intracellular side of the transmembrane domain that are shifted by one turn of the α-helix.

Pentasymmetric protein assemblies are not confined to Cys-loop receptors but frequently occur elsewhere in Nature, for instance in mechanosensitive ion channels, such as MscL, or bacterial toxins, such as Shiga toxin B. Indeed, symmetry-adapted inhibitors for the latter have been developed that show extremely high avidity (picomolar) due to their polyvalency [36]. More recently, polycationic blockers of voltage gated potassium channels based on a four-fold symmetric calixarenes [36] and a blocker of the heptameric Anthrax PA channel based on seven-fold symmetric β-cyclodextrin [37] have been introduced. This underscores that symmetry considerations hold considerable promise for the development of new pharmacophores. Given the prevalence of symmetric protein assemblies in Nature, it seems likely that many symmetry adapted agonists, antagonists and blockers will emerge in future years.

In conclusion, we have identified a new potent blocker of GABA_A_ receptors through rational design rather than a massive screening effort. Our work demonstrates that symmetry considerations can contribute to the pharmacology of Cys-loop receptors. The application of other five-fold-symmetric molecular platforms including some shown in Fig. 1 to the development of high-affinity ligands for GABA_A_ receptors as well as other five-fold symmetric ion channels such as mechanosensitive channels is under active investigation and will be reported in due course.
